# Urban weather dataset for building energy simulations: Data collection and EPW file generation for Torino, Italy (2014–2023)

**DOI:** 10.1016/j.dib.2025.111708

**Published:** 2025-05-27

**Authors:** Ali JahaniRahaei, Massimo Milelli, Giacomo Chiesa

**Affiliations:** a Department of Architecture and Design, Politecnico di Torino, Viale Mattioli 39, 10125 Torino, Italy; b CIMA Foundation, Via A. Magliotto 2, 17100 Savona, Italy

**Keywords:** Urban climate, EPW files, Building energy simulations, Torino weather data, TMY, UHI effect

## Abstract

This dataset provides urban climate data collected from seven weather stations in Torino, Italy, spanning the years 2014 to 2023. The weather stations, operated by ARPA Piemonte, measure key meteorological variables, including air temperature, relative humidity, wind speed and direction, atmospheric pressure, and solar radiation. The data, initially in CSV format, were processed using Python to clean and merge the variables, computing missing parameters such as splitting global irradiation into its components and generating an hourly dataset for each year. Subsequently, a Typical Meteorological Year (TMY) was generated following the ISO 15927-4 standard. Missing data points were estimated using various interpolation and statistical methods to ensure data completeness. Finally, the data were converted into EnergyPlus Weather (EPW) format using custom Python scripts. This dataset serves as a crucial resource for urban climate studies and building energy simulations. It is especially valuable for assessing urban heat island (UHI) effects, supporting the generation of accurate weather files for simulation purposes, and enabling the refinement of design choices in urban planning. The data pipeline can be applied to other cities with weather stations and can also be used in future updates for Torino.

Specifications Table

**Table:** 

Subject	Earth & Environmental Sciences
Specific subject area	Urban climate data for building simulations, focusing on UHI and UDI effects in Turin, Italy.
Type of data	Climate simulation data EPW (EnergyPlus Weather file)
Data collection	The raw weather data were measured by seven weather stations in Torino, operated by Arpa Piemonte. The stations’ sensors recorded air temperature, relative humidity, wind speed and direction, atmospheric pressure, and solar radiation. Data were collected in CSV format and processed using Python (pandas library) to clean, structure, and convert the data into EPW files following the ISO 15927-4 methodology for typical meteorological year (TMY) generation.
Data source location	Torino, To, Piedmont, Italy Weather stations: Venaria La Mandria: (45°10’30’’, 7°33’33’’) Bauducchi: (44°57’36’’, 7°42’31’’) Giardini Reali: (45°04’22’’, 7°41’35’’) Reiss Romoli: (45°06’45’’, 7°40’15’’) Via Della Consolata: (45°04’33’’, 7°40’42’’) Alenia: (45°04’47’’, 7°36’39’’) Caselle: (45°18’05’’, 7°64’80’’)
	
Data accessibility	Repository name: Zenodo Data identification number: 10.5281/zenodo.14905721 Direct URL to data: 10.5281/zenodo.14905721

## 1 Value of the Data

• **Provides Urban Climate Data for Building Simulations**
  These datasets offer urban-scale meteorological information, enabling more accurate building energy simulations rather than using rural reference databases. Researchers and professionals can use the data to analyze urban heat island (UHI) and urban dry island (UDI) effects, replacing rural airport-based climate files commonly used in energy modeling. This possibility will increase the feasibility of urban simulation results, improving the possibility of suggesting performative design optimizations.
• **Enables the Generation of Location-Specific EPW Files**
  The processed data are formatted as EnergyPlus Weather (EPW) files, facilitating direct integration into building performance simulation tools. This allows architects, engineers, and urban planners to conduct climate-responsive design evaluations based on real urban weather conditions and not limited to rural reference data.
• **Supports Research on Urban Climate and UHI Effects**
  The dataset allows for comparative studies between urban and suburban climate conditions. Researchers can use it to investigate microclimatic variations, validate climate models, or assess long-term urban climate trends. This possibility also allows professionals to simulate and analyze UHI countermeasures.
• **Facilitates Future Climate Adaptation and Policy Development**
  Urban climate data are critical for developing sustainable urban policies. This dataset supports policymakers in understanding the impact of urbanization on local climates and in designing strategies to mitigate extreme heat events and the UHI phenomenon in general.

## 2 Background

The motivation behind compiling this dataset stems from the need for accurate urban climate data for building energy simulations and urban heat island (UHI) studies. Traditionally, building energy modeling relies on weather files derived from rural meteorological stations, such as airport weather data, which fail to represent the microclimatic variations within urban environments. To address this gap, we processed and formatted data collected from seven meteorological stations in Torino to create location-specific EnergyPlus Weather (EPW) files

The dataset includes key environmental variables such as air temperature, relative humidity, wind speed and direction, atmospheric pressure, and solar radiation, recorded over multiple years (2014–2023).

This dataset complements an original research article that analyzes urban and suburban climate differences in Torino. Making the processed data openly available allows researchers, architects, and urban planners to conduct more precise building performance simulations and urban climate studies.

### 2.1 Novelty

Despite the availability of raw meteorological data from public sources such as ARPA Piemonte, no standardized or ready-to-use urban weather files existed for the city of Torino prior to this work. Researchers and practitioners had to rely on outdated or rural-based datasets, such as those from airport stations – see the EnergyPlus EPW freely available database [1], or commercial sources, such as the Meteonorm tool [2] – or monthly-average standard reference data for building performance certifications, such as the UNI 10349-1 standard in the Italian case. All do not reflect the microclimatic variability within the urban environment. The process of generating accurate and simulation-ready urban weather files with an hourly definition and freely available involves substantial technical challenges, including accessing and managing dozens of fragmented CSV files across multiple years and variables, cleaning inconsistent data formats, elaborating available data to compute all the required fields to support a building dynamic simulation flow, such as the EnergyPlus one, and addressing missing or erroneous entries. This dataset is the result of a structured and automated data pipeline that integrates cleaning, statistical imputation, and TMY generation in line with ISO 15927-4. By providing EPW files for multiple urban and suburban stations in Torino, this work enables, for the first time, reliable building energy simulations and urban climate studies based on detailed intra-city climatic differences. The dataset fills a critical gap for professionals and researchers working in architecture, engineering, and environmental sciences, offering a scalable and transferable methodology for future urban datasets, allowing in future to easily maintain the database upgraded and to expand the approach to the other available meteorological stations at the national and upper national level.

This dataset is designed to serve not only researchers but also architects, engineers, and urban planners who require accurate and up-to-date climate data for performance-based design. Its availability in EPW format ensures immediate compatibility with widely used simulation tools such as EnergyPlus and its interfaces, e.g. DesignBuilder and Rhino-Grasshopper plugins, and BIM-based energy analysis environments. By eliminating the technical burden of data cleaning and formatting, the dataset lowers the barrier to entry for professionals seeking to incorporate urban climate responsiveness into their workflows. Moreover, the open-access publication of the dataset enhances transparency, supports educational use, and enables future extensions or replications in other urban contexts. This broad applicability underlines the dataset’s role as an enabling resource for data-driven decision-making in the built environment.

## 3 Data Description

The dataset is available in the repository and contains seven separate TMY files, each named in the format *“TMY_{station name}.epw”*. These EPW files correspond to the following weather stations: Bauducchi, Giardini Reali, Reiss Romoli, Via Della Consolata, Alenia, Venaria la Mandria, and Caselle. Each file represents the Typical Meteorological Year (TMY) for the respective station.

## 4 Experimental Design, Materials and Methods

The development of this dataset involved a comprehensive and technically detailed process aimed at converting raw, heterogeneous climate records into standardized and simulation-ready EPW files, compliant with ISO 15927-4 guidelines. While raw meteorological data were accessible from ARPA Piemonte, the transformation into usable weather files required addressing several challenges, including data fragmentation, missing values, and the absence of a unified format. The workflow followed a structured pipeline composed of the following main steps:

• Data Acquisition
• Data Cleaning and Merging
• Missing Data Treatment
• Solar Radiation Decomposition
• TMY Generation
• EPW File Construction

This structured and repeatable pipeline allows for reliable, high-resolution urban weather datasets to be created from publicly available measurements. Its novelty lies in its focus on urban microclimates, automation of the TMY generation process, and direct applicability in simulation tools used across architecture, urban planning, and environmental engineering disciplines.

### 4.1 Data acquisition and processing phases

**Fig. 1:**
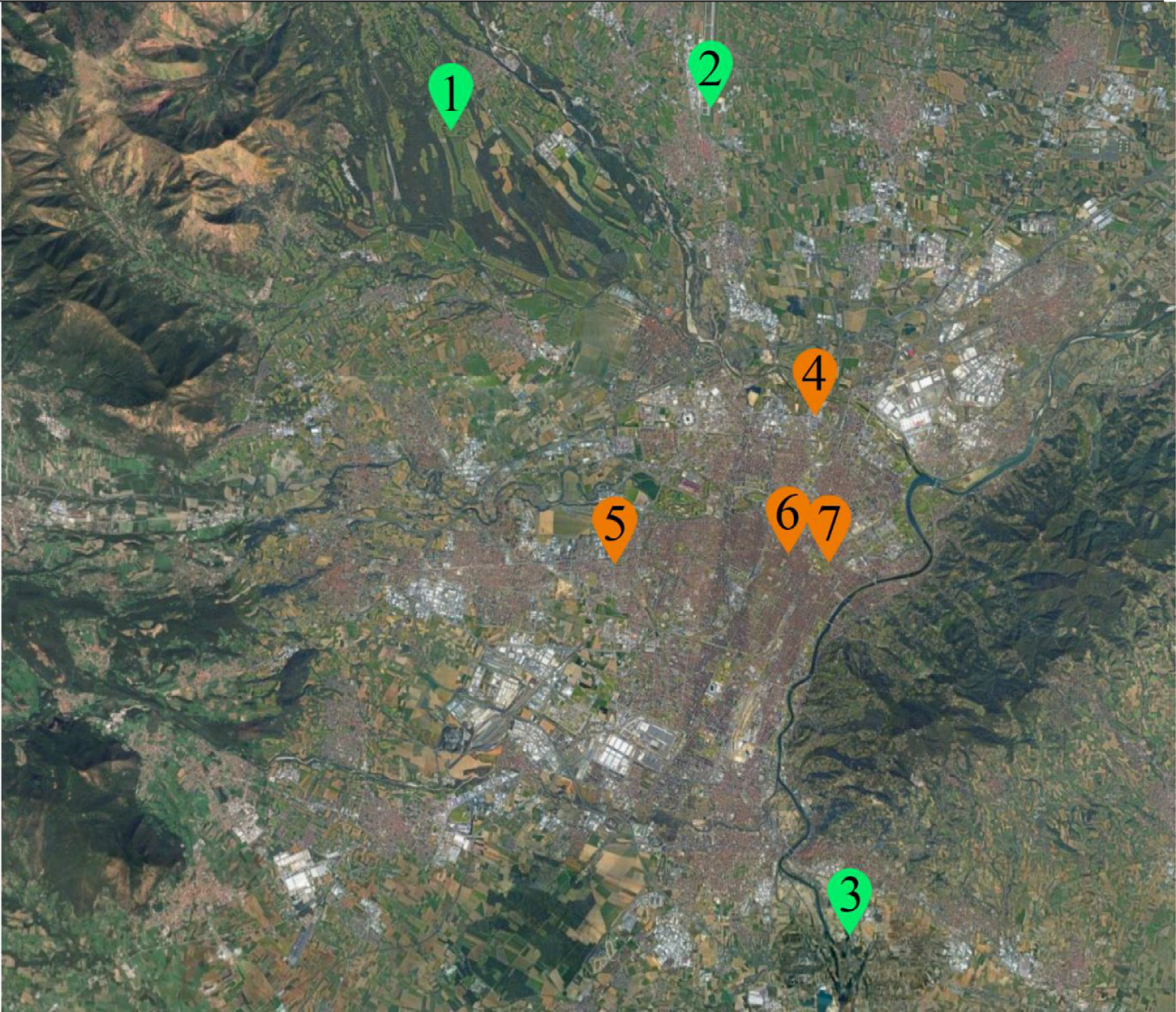
Position of weather stations (City of Turin). Suburban stations (green): 1-Venaria La Mandria, 2- Caselle, 3- Bauducchi. Urban stations (orange): 4- Reiss Romoli, 5- Alenia, 6-Via della Consolata, 7- Giardini reali. Stations 6 and 7 are in the city center, one representative of dense building textures, the second of green areas in the center; stations 4 and 5 are respectively in the boundary between urban and suburban conditions – a commercial high-traffic road, and in the passage between main urban areas and their conurbations.

The dataset was created using meteorological data from seven weather stations in Torino (see Fig. 1), operated by Arpa Piemonte.

The collected variables include:

• Air temperature (°C)
• Relative humidity (%)
• Wind speed (m/s) and wind direction (°)
• Atmospheric pressure (hPa)
• Global solar radiation (W/m²)

Table 1 details the sensors’ specifications and correlated measuring ranges and accuracies.

**Table 1:** The weather station sensors, including manufacturers, models, measurement ranges, errors and resolutions.

Sensor Type	Mode	Manufacturer	Measurement Range	Error Margin	Resolution
Barometer	BA20	CAE S.p.A.	600–1100 hPa	± 0.5 hPa (–10 to 50 °C)	0.1 hPa
				± 1.5 hPa (–20 to 60 °C)	
				± 2 hPa (–40 to 60 °C)	
Anemometer	VV200	CAE S.p.A.	Up to 50 m/s	± 0.07 m/s or 1%	0.05 m/s
Wind Vane	DV200	CAE S.p.A.	0 to 360°	±2.8°	0.35°
Thermometer (Air)	TA20AS	CAE S.p.A.	–40 to +60 °C	± (0.15 + 0.002 × |t|)°C	0.02 °C
Thermo-hygrometer	TU20AS	CAE S.p.A.	–40 to +60 °C	Temp: ± 0.20 °C	Temp: 0.02 °C
			0–100 % RH	Humidity: ±2 % RH	RH: 0.124 %
Pyranometer	CMP 6	Kipp & Zonen	310–2800 nm	± 5 % (daily totals)	1 W/m^2^
Rain Gauge	PG10	CAE S.p.A.	0 to 1000 mm/h	± 3 % (<800 mm/h)	0.1 mm
				± 5 % (800–1000 mm/h)	

For each year from 2014 to 2023, the raw data was downloaded as CSV files directly from Arpa Piemonte’s database. Since the data for each variable was stored in separate files, an automated Python script (using Pandas and NumPy) was developed to:

• Extract the relevant data for each variable from all available years.
• Clean the dataset by handling missing values, removing erroneous data points, and standardizing timestamps.
• Merge the individual variable files into a single dataset containing all meteorological parameters for each station.

Additionally, the EPW file requires a larger set of variables requiring to compute, from existing data, missing fields. Concerning solar radiation, global horizontal values have been split into Diffuse Horizontal Irradiation and Direct Normal Irradiation by exploiting the corresponding Python PREDYCE library EPW compiler expression based on the Boland model [3] - See also the article correlated to this Data in Brief paper for additional details [4]. The same library is also used to compute the Horizontal Infrared Radiation Intensity.

### 4.2 Creating the TMY file

To generate a usable EPW file, raw 10-year data are analysed to generate a Typical Meteorological Year (TMY) – Generated following the ISO 15927-4 standard, which selects representative months based on long-term statistical distributions. A Python script was developed and used to process the dataset, extract key statistical metrics (mean, standard deviation, cumulative distribution), and apply the statistics method to identify the most representative months for constructing the TMY.

### 4.3 Handling missing data

Since some variables had occasional missing values, different methods were applied for imputation – see also [5]: linear interpolation was used for short-term gaps (e.g., missing hours), while wind speed and solar radiation were adjusted using correlations between different stations where data was available.

### 4.4 Generating the EPW File

Once the final TMY dataset was constructed, a Python script was used to convert it into EPW format, ensuring compatibility with building energy simulation tools (e.g., EnergyPlus, DesignBuilder). This included:

• Formatting the data according to EnergyPlus EPW specifications.
• Assigning metadata such as location coordinates, time zone, and altitude to construct the EPW file header.
• Verifying the output by running the EnergyPlus simulation.

The resulting EPW files provide a reliable urban weather dataset for building energy simulations in Torino, addressing the lack of urban climate data for architects and urban planners.

### 4.5 Discussing the meaning of the urban-generated EPW

**Fig. 2:**
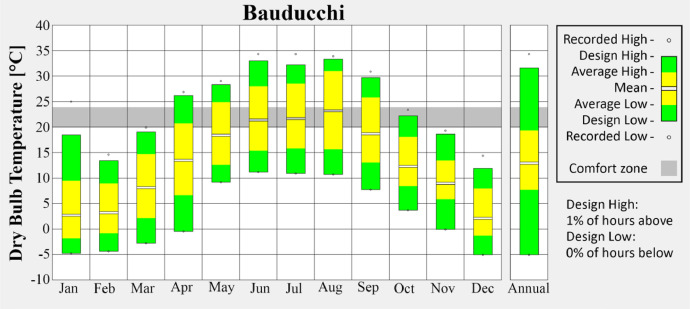
Monthly average temperature of Bauducchi weather station.
• Caselle (Fig. 3) is located near the Turin Airport, serving as a reference for suburban climatic conditions influenced by infrastructure and open surroundings.

Among the seven weather stations included in the dataset, four were selected for graphical representation in order to illustrate the diversity of urban and suburban climatic conditions across Torino. The selected stations reflect distinct urban typologies:

• Bauducchi (Fig. 2) lies in a suburban open field context, representing areas with low building density and minimal anthropogenic influence.

**Fig. 3:**
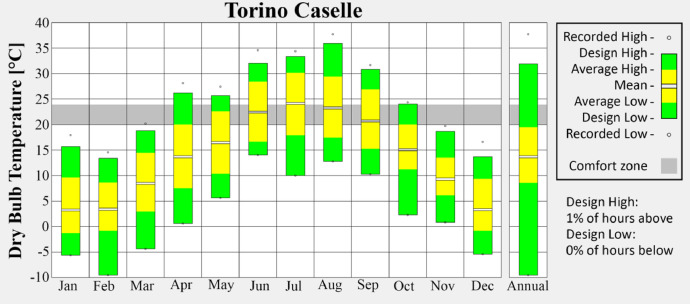
Monthly average temperature of Caselle weather station.
• Via della Consolata (Fig. 4) represents the dense urban city center, characterized by compact building layouts and limited vegetation.

**Fig. 4:**
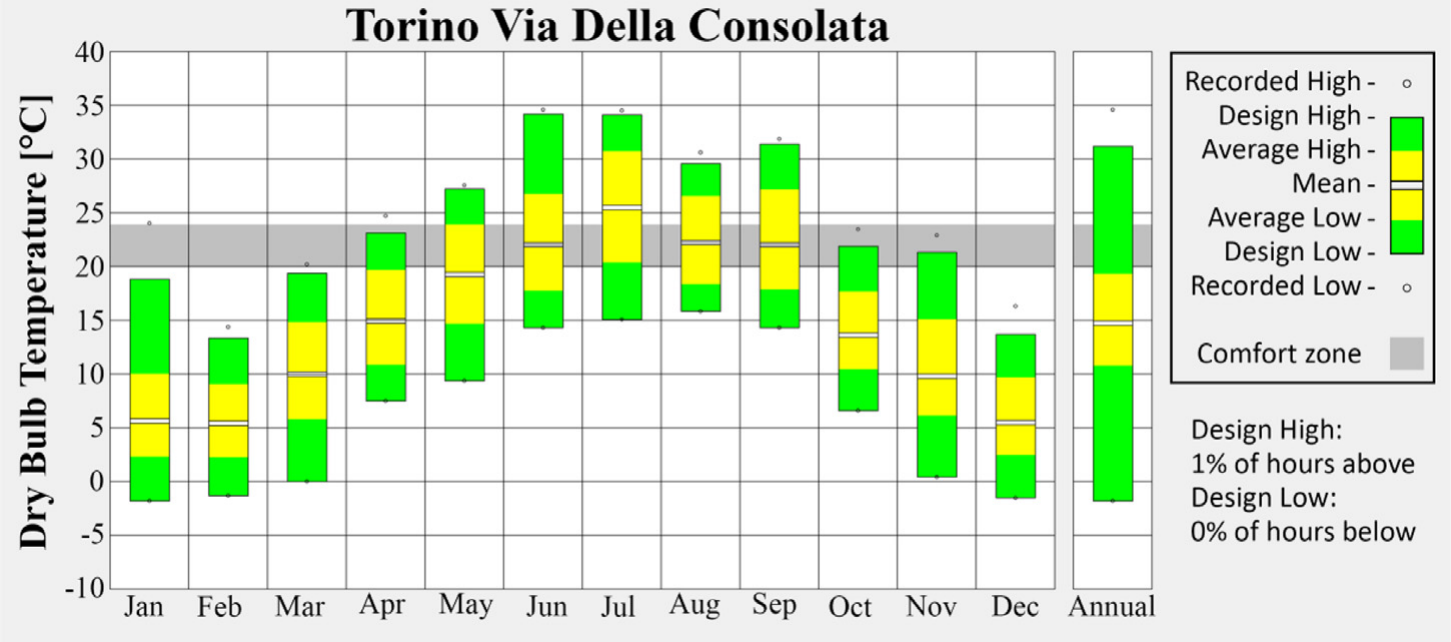
Monthly average temperature of Via della Consolata weather station.
• Giardini Reali (Fig. 5) corresponds to an urban context with significant green areas, capturing the moderating influence of vegetation within the city.

**Fig. 5:**
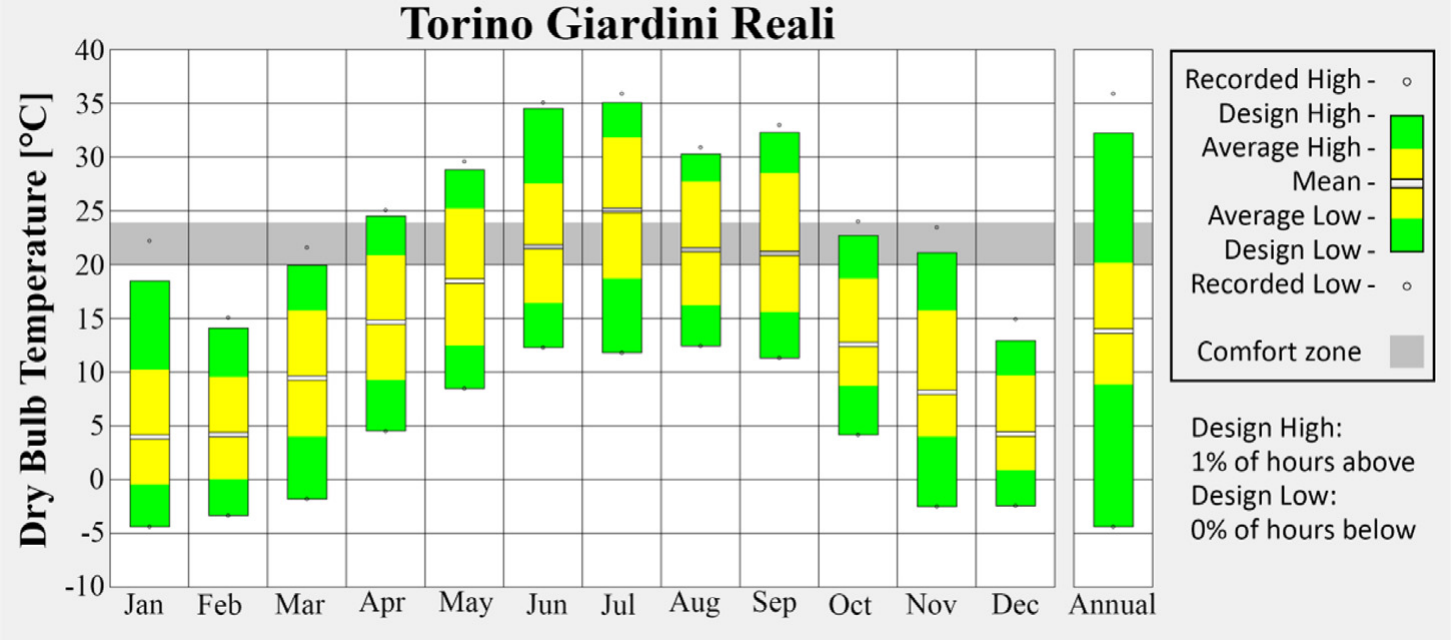
Monthly average temperature of Giardini Reali weather station.

These visualizations highlight seasonal variations and provide insight into the urban climate trends captured in the dataset. Figures are produced via the Climate Consultant tool [6].

Additionally, the following Tables 2 to 4 report respectively: Table 2. the monthly and yearly average dry bulb temperatures [°C], Table 3. the monthly and yearly average wind speeds [m/s], and Table 4. the daily monthly average global horizontal irradiation [kWh/m^2^]. To contextualize the obtained results, the tables also include reference values from the Italian standard UNI 10349-1 [7], which is widely used in the field of building energy performance assessments and for the official Energy Performance Certification procedures. It should be emphasized that the UNI 10349-1 dataset is based on earlier climatic records, with the latest release dating back to 2016 and reflecting a reference period prior to the time frame considered in this study. Moreover, the standard is primarily designed for quasi-steady state calculation methods, relying on monthly averages and generalized assumptions, and is therefore not suited for dynamic simulation approaches that require detailed hourly input. The Bauducchi station is the designated reference site for the Turin area within the standard.

**Table 2:** Monthly and yearly average dry bulb temperatures [°C]

	Station	JAN	FEB	MAR	APR	MAY	JUN	JUL	AUG	SEP	OCT	NOV	DEC	Annual Avg.
Dry Bulb Temperature [°C]	Bauducchi	2.7	3.2	8.1	13.5	18.4	21.4	21.7	23.2	18.7	12.3	9.0	2.1	12.9
	Caselle	3.3	3.4	8.5	13.7	16.5	22.5	24.2	23.3	20.7	15.0	9.3	3.3	13.7
	Alenia	4.4	4.7	9.5	14.7	16.5	21.3	27.3	24.0	19.1	15.5	9.0	5.8	14.4
	Giardini Reali	3.9	4.2	9.4	14.7	18.5	21.7	25.1	21.4	21.1	12.5	8.1	4.2	13.8
	Reiss Romoli	4.7	4.8	9.5	14.4	17.2	21.5	27.5	21.5	19.5	13.1	10.1	5.6	14.2
	Via Della Consolata	5.6	5.4	10.0	14.9	19.2	22.0	25.5	22.2	22.0	13.6	9.8	5.5	14.7
	Venaria La Mandria	2.4	3.0	7.7	12.9	17.4	20.6	25.9	22.4	19.4	11.4	8.3	3.4	13.0
	UNI 10349-1	1.3	3.2	8.4	12.0	18.1	22.2	23.7	22.7	19.2	12.4	6.9	2.7	12.8

**Table 3:** Monthly and yearly average wind speeds [m/s]

	Station	JAN	FEB	MAR	APR	MAY	JUN	JUL	AUG	SEP	OCT	NOV	DEC	Annual Avg.
Wind Speed [m/s]	Bauducchi	1.2	1.3	1.7	1.6	1.5	1.4	1.4	1.3	1.2	1.1	1.0	0.7	1.3
	Caselle	1.7	1.9	2.3	2.2	2.2	2.1	2.1	2.0	1.8	1.6	1.6	1.2	1.9
	Alenia	1.9	1.9	2.2	2.0	2.0	1.9	2.0	1.8	1.7	1.6	1.6	1.5	1.9
	Giardini Reali	1.0	1.0	1.2	1.3	1.3	1.2	1.3	1.1	1.0	0.9	0.9	0.8	1.1
	Reiss Romoli	1.5	1.7	2.0	1.9	1.9	1.9	1.9	1.9	1.6	1.4	1.4	1.2	1.7
	Via Della Consolata	1.2	1.3	1.5	1.6	1.6	1.5	1.6	1.4	1.3	1.1	1.1	1.0	1.4
	Venaria La Mandria	1.0	1.0	1.4	1.0	0.8	0.7	0.7	0.6	0.8	0.6	0.9	0.9	0.9
	UNI 10349-1	1.3	1.3	1.6	1.9	1.9	1.6	1.6	1.4	1.1	1.2	1.5	0.9	1.4

**Table 4:** Daily monthly average global horizontal irradiation [kWh/m^2^].

	Station	JAN	FEB	MAR	APR	MAY	JUN	JUL	AUG	SEP	OCT	NOV	DEC
**Global Horizontal Irradiation [kWh/m^2^]**	Bauducchi	1.7	2.0	4.0	5.5	6.0	6.6	6.3	6.7	4.5	2.5	1.2	1.4
	Caselle	1.5	1.9	3.8	5.0	5.5	6.3	6.3	5.1	4.1	2.3	1.1	1.3
	Alenia	1.7	2.1	4.1	4.7	5.4	6.2	6.5	6.1	4.2	2.5	2.2	1.3
	Giardini Reali	1.3	1.8	3.7	4.7	5.2	5.7	6.2	4.6	4.3	2.2	1.5	1.0
	Reiss Romoli	1.7	1.9	3.9	4.9	5.7	6.1	6.4	5.1	4.1	2.5	1.2	1.2
	Via Della Consolata	1.5	1.8	3.5	4.3	4.9	5.3	5.6	4.4	4.0	2.2	1.9	1.4
	Venaria La Mandria	1.5	1.9	3.8	4.6	5.4	5.8	6.2	5.8	4.1	2.3	1.3	1.3
	UNI 10349-1	1.3	2.1	3.3	4.4	5.5	6.3	6.7	5.6	4.1	2.5	1.3	1.1

Similarly, it can be mentioned that in existing EPW databases, giving professionals and researchers access to data feeding hourly-detailed simulations, available data are correlated only to rural, generally not upgraded, datasets: e.g. the EnergyPlus weather source [1] gives access to two versions of the Torino Caselle (airport) weather elaborated TMY, one referring to the IGDG (Italian climatic “Gianni de Giorgio”) database referring to data of the 1951–1970 period of record, and the second to the IWEC (International Weather for Energy Calculation) by ASHRAE based on © 2001 data – see also [8,9]; while the Meteonorm available datapoints (2000–2019), with at least partial data retrieved from meteorological stations, refer to Caselle (airport) and to Bric della Croce (a hill point without irradiation measurements). The newly proposed database is, hence, a visible upgrade to the currently available ones.

## Limitations

The EPW files in this dataset were generated using measured data from urban weather stations, with sensors available for key meteorological variables. Urban stations, with the exclusion of the Giardini Reali one, are located outside urban canyons, positioned on the rooftop. Data users may eventually adopt morphing approaches to scale wind speeds within local canyons. Additionally, some missing fields were imputed using methods described in the previous section. However, certain variables, such as illuminance, are missing and have been filled with the corresponding predefined missing values’ codes for EPW files [10]. It is important to note that these missing fields do not affect the building energy simulations, and the EPW files can be used without issues in EnergyPlus simulations – see again [10].

## Ethics Statement

The authors confirm that they have read and followed the ethical requirements for publication in *Data in Brief*. This work does not involve human subjects, animal experiments, or any data collected from social media platforms. The dataset consists of meteorological measurements obtained from publicly available weather stations operated by Arpa Piemonte, ensuring compliance with ethical and data-sharing guidelines

## CRediT Author Statement

**Ali JahaniRahaei:** Methodology, Software, Data Curation, Writing – Original Draft. **Massimo Milelli:** Conceptualization, Methodology, Data Curation, Writing – Original Draft. **Giacomo Chiesa:** Conceptualization, Methodology, Writing – Original Draft, Review & Editing, Supervision and Funding Acquisition

## Data Availability

ZenodoTorino-EPW (Original data)
